# Characterizing infraocclusion in primary molars: prevalence, accompanying findings, and infraocclusion severity and treatment implications

**DOI:** 10.1186/s12903-024-04428-x

**Published:** 2024-06-05

**Authors:** Beyza Ballı Akgöl, Nilüfer Üstün, Merve Bayram

**Affiliations:** 1 School of Dentistry, Department of Pediatric Dentistry, Antalya Bilim University, Antalya, Turkey; 2 School of Dentistry, Department of Pediatric Dentistry, Istanbul Medipol University, Istanbul, DDS 34093 Turkey

**Keywords:** Infraocclusion, Prevalence, Severity, Treatment types, Children

## Abstract

**Background:**

This manuscript investigates the prevalence, classification, accompanying findings, and treatment modalities associated with infraoccluded primary molars. The aim of this study categorizing primary molars based on the severity of infraocclusion and assessing their respective treatment interventions across different severity groups.

**Methods:**

The classification, treatment types, accompanying findings, and the condition of succeeding premolars of infraoccluded molars were documented. Chi-square tests, including Fisher’s Exact Chi-square test, Fisher Freeman Halton Exact Chi-square test, and One Sample Chi-square test, were conducted. The predetermined significance level was less than 0.05.

**Results:**

The study population consisted of 3132 subjects aged 3 to 15 years, with a prevalence of 4.3% for infraocclusion. Infraocclusion typically manifests between 6 and 9 years of age and predominantly affects mandibular primary molars. Treatment interventions varied based on infraocclusion severity, with more invasive procedures required for severe cases. Accompanying findings associated with infraocclusion include adjacent teeth tipping, significant deviation in midline shifts towards the affected side and increased caries. Additionally, succeeding premolar agenesis was observed in 2% of infraoccluded molars, with extraction rates higher in cases where the successor tooth was mesially or distally located.

**Conclusions:**

The study offers novel insights to dental practitioners concerning the severity and distribution of treatment interventions for infraocclusion. It suggests that more severe cases may necessitate more invasive procedures, with the aim of enhancing patient outcomes through timely intervention and personalized therapeutic strategies.

## Introduction

Infraocclusion, a clinical manifestation characterized by a tooth positioned beneath the occlusal plane, may manifest in both primary and permanent dentition [1]. Various terms such as impaction, submerged, ankylosed, incomplete eruption, secondary retention, reimpaction, and submerging have been employed in the literature to describe this phenomenon [1, 2]. The multitude of terminologies arises from the uncertain etiology underlying infraocclusion [2].

The literature reports a prevalence range of infraocclusion in primary teeth spanning from 1.3 to 38.5% [3–7]. Remarkably, infraocclusion manifests at a frequency approximately ten times higher in primary dentition compared to permanent dentition [8, 9]. While historical data often identified mandibular second molars as the most commonly affected teeth [10], recent research suggests a reversal, with infraocclusion predominantly occurring in the first primary mandibular molars [11–14]. The substantial variability in prevalence rates and affected teeth across studies is attributed to differences in sample sizes, diagnostic methodologies, age demographics, inclusion/exclusion criteria, and ethnic diversities [15–17]. Although infraocclusion may manifest as early as around 3 years of age [9], definitive diagnosis typically occurs between 6 and 11 years [18, 19]. Furthermore, the incidence of infraocclusion exhibits no discernible gender disparity, and no significant discrepancy is noted between its occurrence on the right versus left sides of the arch [1, 15, 19].

Infraoccluded molars have the potential to precipitate various orthodontic and periodontal complications. These may encompass adjacent teeth tipping, space loss, lateral open bite, diminished arch length, midline deviation shifting towards the affected side, and extrusion of antagonist teeth. Additionally, infraocclusion may contribute to periodontal issues such as apically positioned gingival margins, periodontal diseases, and increased caries susceptibility [2, 15, 20, 21]. Detection of severe infraocclusion can exacerbate orthodontic challenges, complicating the application of corrective forces. Dental anomaly patterns (DAP), as conceptualized by Sheldon Peck [22], characterize biologically associated dental anomalies wherein certain dental abnormalities co-occur at a frequency significantly exceeding that expected by random chance alone. Infraocclusion which is considered among the DAP, predominantly observed in deciduous teeth, correlates with various measurable or visually identifiable conditions. These include tooth absence, microform teeth (e.g., peg-shaped lateral incisors), generalized or localized tooth-size reduction, generalized or localized delays in tooth formation and eruption, palatal displacement of canines, maxillary canine-first premolar transposition, mandibular lateral incisor-canine transposition, and distal angulation of unerupted mandibular second premolars [22].

The prompt recognition and management of infraocclusion observed in children during the primary and mixed dentition phases are imperative to enhance treatment efficacy. Identification of an infraoccluded tooth warrants attention, as it signifies an increased susceptibility of other primary teeth to similar infraocclusive conditions. It is crucial to document infraocclusion relative to neighboring teeth to monitor its progression rate and severity accurately. Utilization of radiographic modalities becomes essential for discerning any impact of infraocclusion on permanent tooth development. Emphasis on oral hygiene education and implementation of protective measures are paramount to mitigate the risk of caries in adjacent dentition linked with infraocclusion. Consequently, precise diagnosis and early intervention facilitate strategic planning of clinical options, thereby minimizing the necessity for future multidisciplinary interventions, including orthodontic, surgical, and prosthetic approaches [23]. This holistic approach underscores the significance of early intervention in optimizing treatment outcomes and long-term oral health.

Awareness of the classification degree of infraocclusion holds paramount importance for dental professionals in guiding appropriate treatment decisions. To date, there is a notable absence of literature addressing the treatment demands associated with infraoccluded primary molars and the severity classification of infraocclusion. The present retrospective study endeavors to fill this knowledge gap by categorizing primary molars based on the severity of infraocclusion and assessing their respective treatment interventions across different severity groups. The study hypothesizes that the severity of infraocclusion in primary molars may significantly influence the required treatment interventions. Moreover, the study aims to investigate the prevalence and accompanying findings associated with infraoccluded primary molars, as well as evaluate the presence, and location distribution of succeeding premolars. This comprehensive approach aims to provide insights into the management strategies for infraocclusion in primary molars and its implications for subsequent dental development.

## Materials and methods

Approval for this study was granted by the Ethics Committee of Istanbul Medipol University (REF:10840098-772.02-E.61,666). Data were retrieved from electronic patient records spanning from 2015 to 2020. A total of 4828 panoramic radiographs underwent retrospective evaluation.

### Study population

This study recruited children attending the department of pediatric dentistry for routine dental care and possessing panoramic radiographs. To delineate the study population, three primary inclusion criteria were established as follows:

i. Inclusion of patients with panoramic radiographs featuring clear and undistorted images.
ii. Enrolment of children spanning the primary, mixed, and permanent dentition phases and exhibiting primary molars.
iii. Inclusion of systemically healthy children.

Exclusion criteria involved subjects with subpar quality panoramic radiographs, systemic diseases, syndromes, cleft lip and palate, disabilities, or undergoing ongoing orthodontic treatment. Among the initial cohort of 4027 subjects, 736 individuals undergoing orthodontic treatment and 159 subjects with systemic diseases, syndromes, or disabilities were excluded. Consequently, a total of 3132 subjects with panoramic radiographs meeting the inclusion criteria were selected for analysis.

### Retrospective data acquisition

Patients were identified using a unique patient protocol number and case number generated from database records to maintain confidentiality. Recorded data included case number, gender, age, and presence or absence of infraocclusion for all subjects. Additionally, the number, jaw, and location of infraoccluded teeth were assessed. Data collection and evaluation were performed independently by two trained examiners (B.B.A and N.U). Interexaminer reliability was assessed by evaluating 100 panoramic radiographs, yielding a kappa value of 0.81.

### Classification of infraoccluded molars

**Fig. 1:**
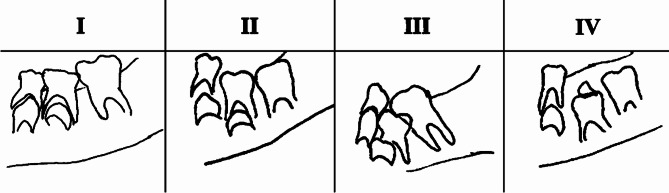
Kjaer classification categories [19]

In this investigation, the classification of primary molar infraocclusion was conducted through direct visual inspection from orthopantomograph, based on the measurement of the distance from the occlusal level of the primary molars to the adjacent teeth in millimeters, following the Kjaer classification system (Fig. 1) [19]:

#### “Group I

Cases with a mild degree of infraposition were classified into Group I. In this group, the level of occlusion of the primary molar was equal to or less than half crown height of the actual primary molar when the occlusal level was compared with the occlusal surface of one or two fully erupted neighbouring teeth.

#### Group II

In cases belonging to Group II, the level of occlusion of the primary molar was half to full crown height below the level of the occlusal surface of one or two fully erupted neighbouring teeth.

#### Group III

Cases with a severe degree of arrest were classified into Group III. The level of occlusion of the primary molar was equal to or more than full crown height below the level of the occlusal surface of one or two fully erupted neighbouring teeth.

#### Group IV

Cases with an extreme degree of arrested eruption were classified into Group IV. The primary molar was found deeply subgingivally retained to such an extent that the occlusal surfaces of the fully erupted neighbouring teeth were located at a distance equal to or more than one and a half crown height of the primary molar compared with the level of the neighbouring teeth.”

### Treatment types of infraoccluded molars

There are no standardized official guidelines for clinicians regarding on the management of infraoccluded primary molars. Data pertaining to treatment modalities previously performed were extracted from individual patient records on a case-by-case basis. The treatment types for infraoccluded molars were categorized into six groups for assessment:

i. No intervention needed, monitor biannually.
ii. Preventive treatment.

 (Glass Ionomer-Based Fissure Sealants, Resin-Based Fissure Sealants, Fluoride Theraphy.

iii. Restorative treatment.

 (Glass Ionomer Cement Restorations, Compomer Restorations, Composite Restorations, Composite Strip Crown Restorations, Composite Onlays, Amalgam Restorations)

iv. Pulpal involvement (Pulpotomy, Pulpectomy).
v. Extraction.
vi. Stainless-steel crowns (SSC) or zirconia crown.

Patients requiring orthodontic space opening following extraction of severely infraoccluded deciduous teeth and spontaneous eruption of impacted premolars were treated in a distinct zone of the hospital. Therefore, they were not included in the study as a treatment group due to the unavailability of data.

### Accompanying findings of infraoccluded molars

Accompanying findings associated with infraoccluded molar were evaluated as follows:

i. Tipping of adjacent teeth.
ii. Supraeruption of opposing tooth.
iii. Increase caries.
iv. Significant deviation in midline shifts towards the affected side.
v. Root resorption.

### The succeeding premolar

The presence or absence of the successor permanent tooth beneath the infraoccluded tooth was documented in a spreadsheet. The positioning of the succeeding premolar was assessed according to the following criteria:

i. Occlusally.
ii. Mesially.
iii. Distally.

### Statistical analysis

Statistical analysis of all data was conducted by a single independent examiner (MB) using IBM SPSS software version 25 (IBM Corporation, Armonk, NY, USA). Descriptive statistical methods including mean, standard deviation, and frequency distributions were employed. Moreover, Chi-square test, Fisher’s Exact Chi-square test, Fisher Freeman Halton Exact Chi-square test, and One Sample Chi-square test were utilized for comparing qualitative data. The significance level was set at < 0.05.

## Results

In this retrospective analysis, panoramic radiography records of 3132 subjects [1646 (%52.6) male, 1486 (%47.4) female] aged between 3 and 15 years (mean age: 8.03 ± 1.89 years) were analyzed. The prevalence of infraocclusion within the study population was determined to be 4.3%. Demographic characteristics of the study cohort and the subgroup with infraocclusion are presented in Table 1.

**Table 1:** Demographic data

Demographic features	Study Population (*n* = 3132)		Infraocclusion population (*n* = 135)	
Age				
Min-Max	3–15		4-12.4	
Mean	8.03 ± 1.89		8.16 ± 1.71	
	**n**	**%**	**n**	**%**
Age Groups				
3 - < 6 years	430	13.7	10	7.4
6 - < 9 years	1725	55.1	85	63.0
9- < 12 years	897	28.6	37	27.4
12 - < 15 years	80	2.6	3	2.2
p			0.001*	
Gender				
Male	1646	52.6	59	43.7
Female	1486	47.4	76	56.3
p			0.143	

One sample chi square test *p < 0.05

A statistically significant difference was observed among the age groups of subjects with infraocclusion (*p* < 0.05). Specifically, the prevalence of infraocclusion was notably higher in the 6–9 years age group compared to all other age groups (*p* < 0.05). Similarly, the incidence of infraocclusion in the 9–12 years age range was significantly greater than that observed in the 3-6- and 12-15-years age groups (*p* < 0.05). Gender distribution among children with infraocclusion did not display a statistically significant difference (*p* > 0.05). Table 2 illustrates the distribution of infraoccluded molar classes across different age categories.

**Table 2:** Distribution of class of infraoccluded molars according to age groups

	3–6 years	6–9 years	9–12 years	12–15 years	*p*
Classification	*n* (%)	*n* (%)	*n* (%)	*n* (%)	
Group I	14 (%87.5)	105 (%80.8)	45 (%93.8)	3 (%100)	0.567
Group II	1 (%6.3)	17 (%13.1)	3 (%6.3)	0 (%0)	
Group III	1 (%6.3)	6 (%4.6)	0 (%0)	0 (%0)	
Group IV	0 (%0)	2 (%1.5)	0 (%0)	0 (%0)	

Fisher’s exact test

No significant difference was found between age groups concerning the distribution of infraocclusion classes (*p* > 0.05). While infraocclusion at Group I and Group II levels was observed across all age groups, infraocclusion at Group IV level was solely identified in subjects aged 6–9 years

Distributions of the number infraoccluded primary molars are shown in Table 3.

**Table 3:** Distributions of the number of primary molars in infraocclusion population (*n* = 135)

		*n*	%	*p*
Number of primary molars in infraocclusion	1	93	68.9	0.001*
	2	30	22.2	
	**≥**3	12	8.9	
Maxilla	0	105	77.8	0.001*
	1	25	18.5	
	**≥**2	5	3.7	
Mandible	0	20	14.8	0.001*
	1	77	57.0	
	**≥**2	38	28.2	
Right side	0	30	22.2	0.001*
	1	95	70.4	
	2	10	7.4	
Left side	0	65	48.2	0.001*
	1	60	44.4	
	**≥**2	10	7.4	
1st primary molar	0	38	28.1	0.001*
	1	67	49.6	
	**≥**2	30	22.2	
2nd primary molar	0	88	65.2	0.001*
	1	32	23.7	
	**≥**2	15	11.1	

One sample chi square test *p < 0.05

Among subjects exhibiting infraocclusion, it was noted that 68.9% had infraocclusion in one tooth, 22.2% in two teeth, and 8.9% in three or more teeth. A statistically significant difference was observed in the distribution of deciduous molars affected by infraocclusion (*p* < 0.05). Specifically, the incidence of infraocclusion in a single molar was significantly higher compared to that in two or more molars (*p* < 0.05). Similarly, the incidence of infraocclusion in two molars was significantly higher than in three or more molars (*p* < 0.05). Furthermore, the incidence of infraocclusion in a single molar in both the maxilla and mandible was significantly higher than in two or more molars (*p* < 0.05). Moreover, the incidence of infraocclusion in one tooth on the right side (70.4%) was significantly higher than the incidence in two teeth (7.4%) (*p* < 0.05). Conversely, the incidence of infraocclusion in two or more teeth on the left side (7.4%) was significantly lower than that of a single tooth (44.4%) (*p* < 0.05). Additionally, the incidence of infraocclusion in a single tooth among first primary molars (49.64%) was significantly higher compared to that in two or more teeth (22.2%) (*p* < 0.05). Similarly, the incidence of infraocclusion in a single tooth among second primary molars (23.7%) was significantly higher than the incidence in two or more teeth (11.1%) (*p* < 0.05).

**Fig. 2:**
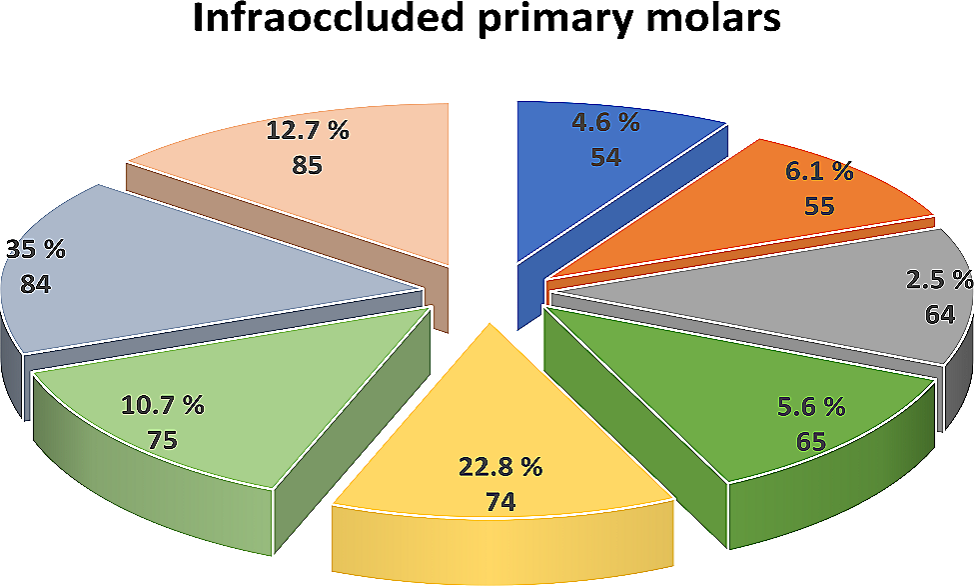
Data regarding primary molars afflicted with infraocclusion

The predominant infraoccluded primary molar observed on the dental arch was the lower right first primary molar, accounting for 35%, followed by the lower left first primary molar at 22.8%, and the lower right second primary molar at 12.7% (Fig. 2).

**Fig. 3:**
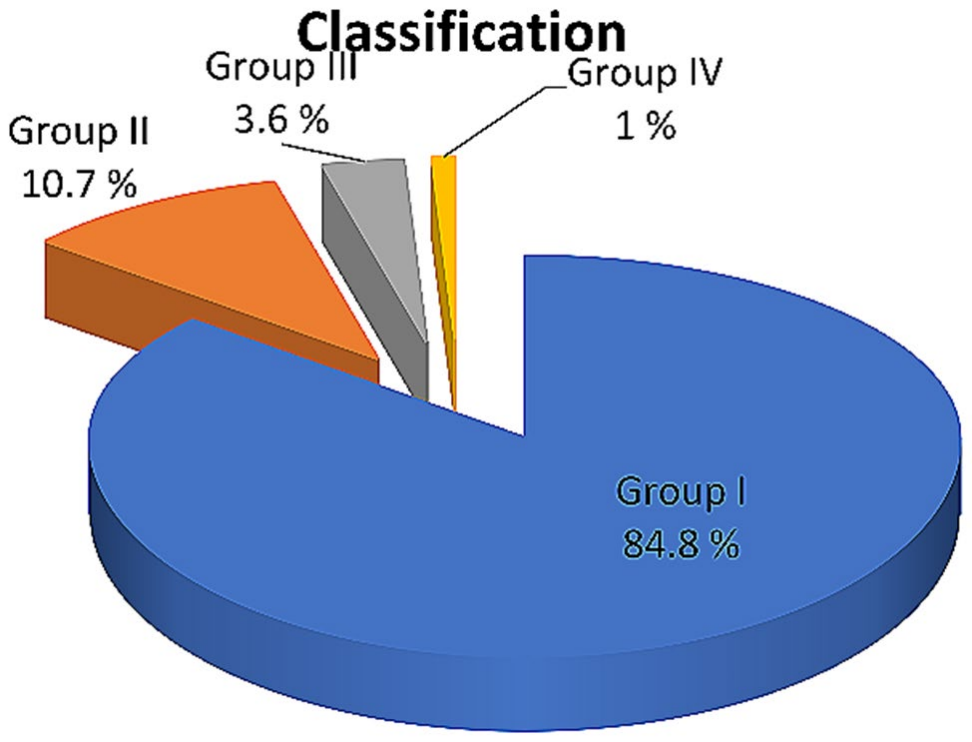
Data regarding classification of infraoccluded primary molars

**Fig. 4:**
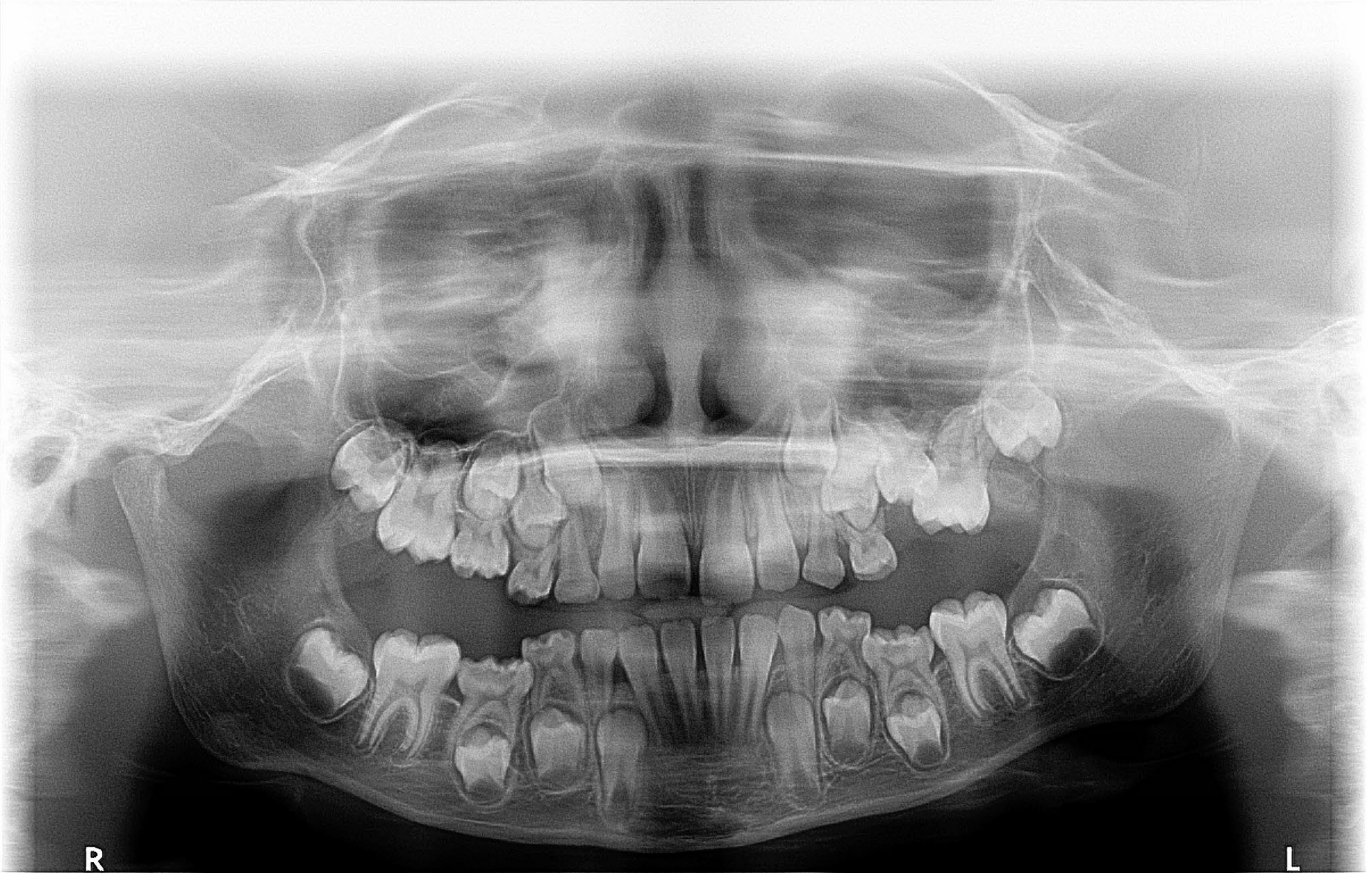
A panoramic radiograph of a female aged 6 years and 7 months revealed tooth numbers 55, 75, and 85 presenting as Group II cases. Meanwhile, tooth number 65 exhibited a severe degree of infraocclusion, classified as a Group III case. It’s worth noting that the succeeding premolar of tooth number 65 was located mesially

**Fig. 5:**
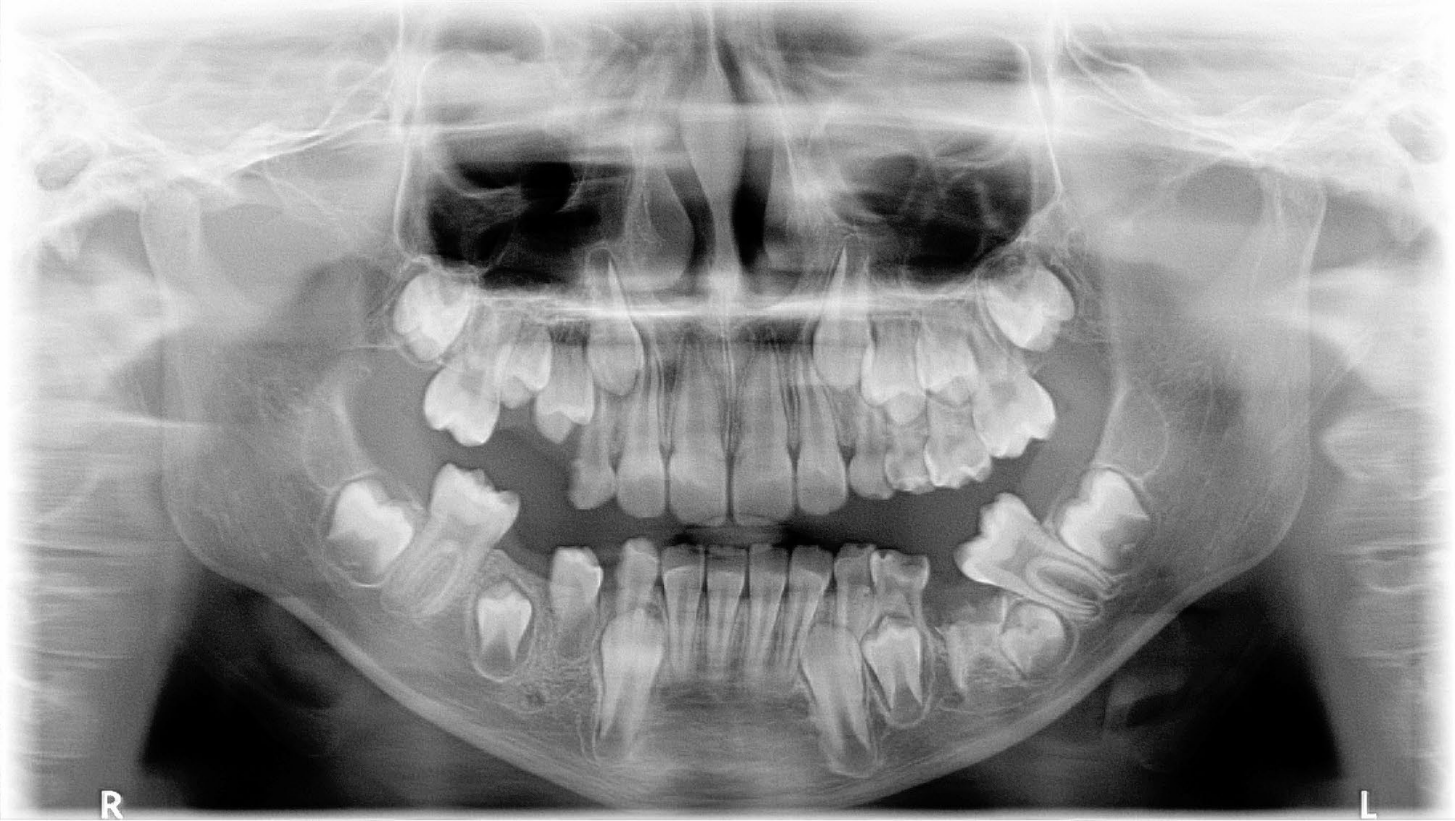
A panoramic radiograph of a girl aged 8 years and 9 months showed tooth number 75 with a severe degree of infraocclusion, classified as a Group III case. Accompanying features included extreme tipping of the adjacent first permanent molar, increased caries, and a significant deviation in midline shift towards the affected side. It is worth noting that the succeeding premolar of tooth number 75 was located distally

**Fig. 6:**
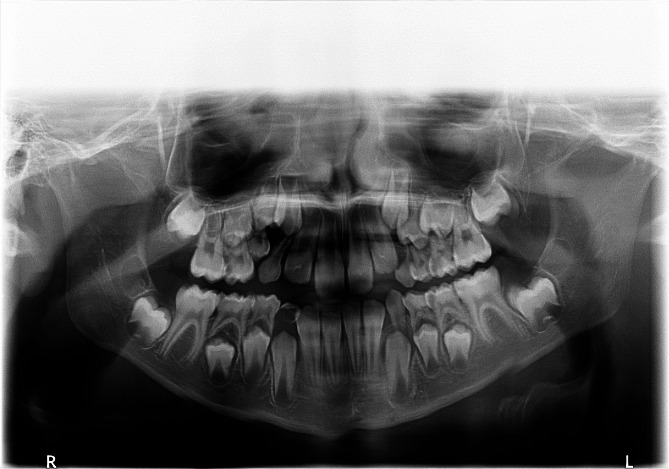
A panoramic radiograph of a female aged 8 years showed tooth number 54 presenting a severe degree of infraocclusion, classified as a Group III case. Tipping of adjacent teeth was observed as an accompanying finding

**Fig. 7:**
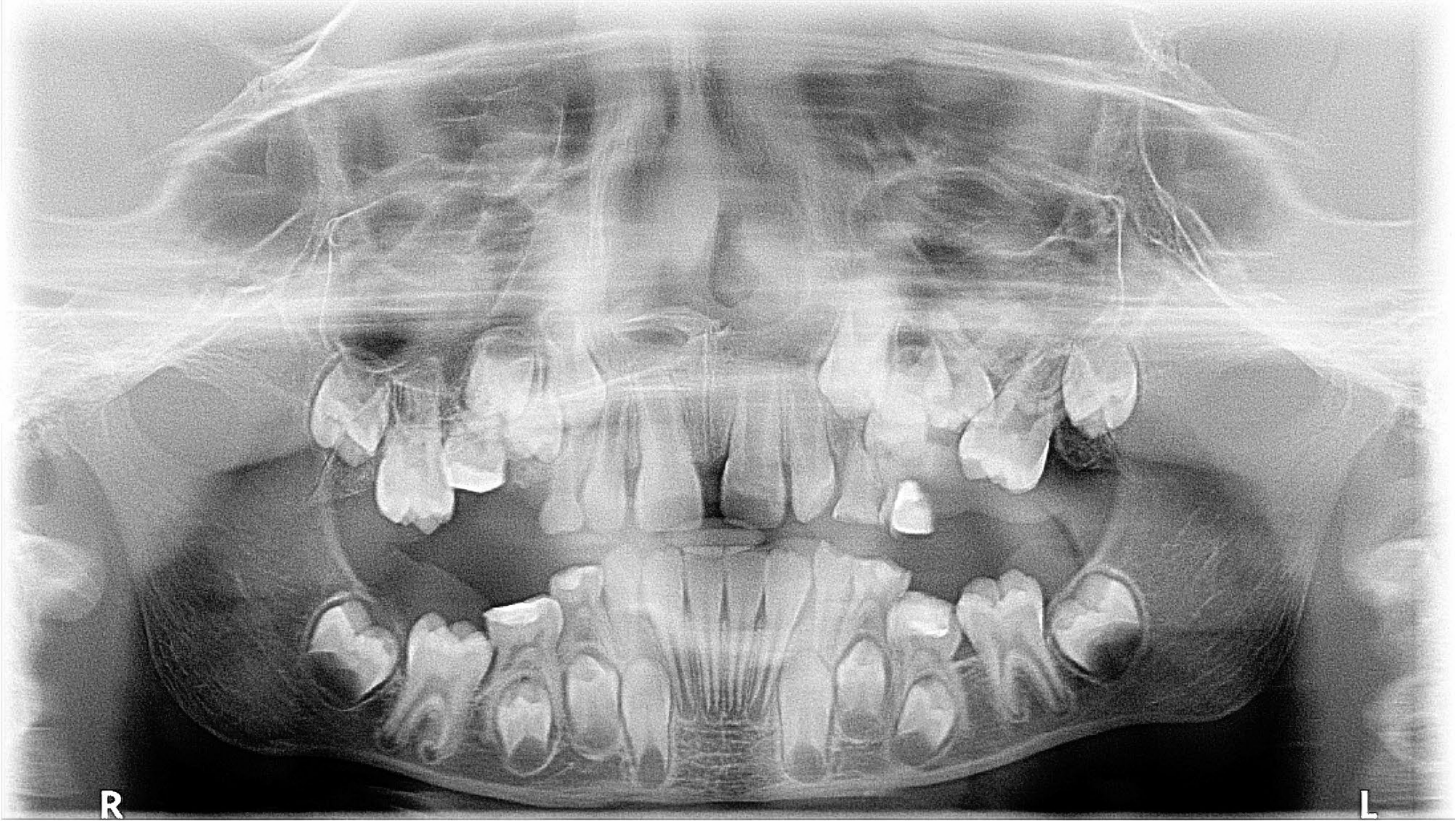
A panoramic radiograph of a male, aged 6 years and 6 months, showed tooth numbers 55, 75, and 85 evaluated as Group II classification, while tooth number 65 presented an extreme degree of infraocclusion classified as a Group IV case. It is worth noting that the succeeding premolar was located mesially

Considering the four classifications of infraocclusion, the majority (84.8%) of affected molars were categorized as Group I infraocclusion, followed by 10.7% classified as Group II, 3.6% as Group III, and 1% as Group IV, representing the most severe category (Fig. 3). Illustrations depicting group categories are provided in Figs. 4, 5, 6 and 7.

**Fig. 8:**
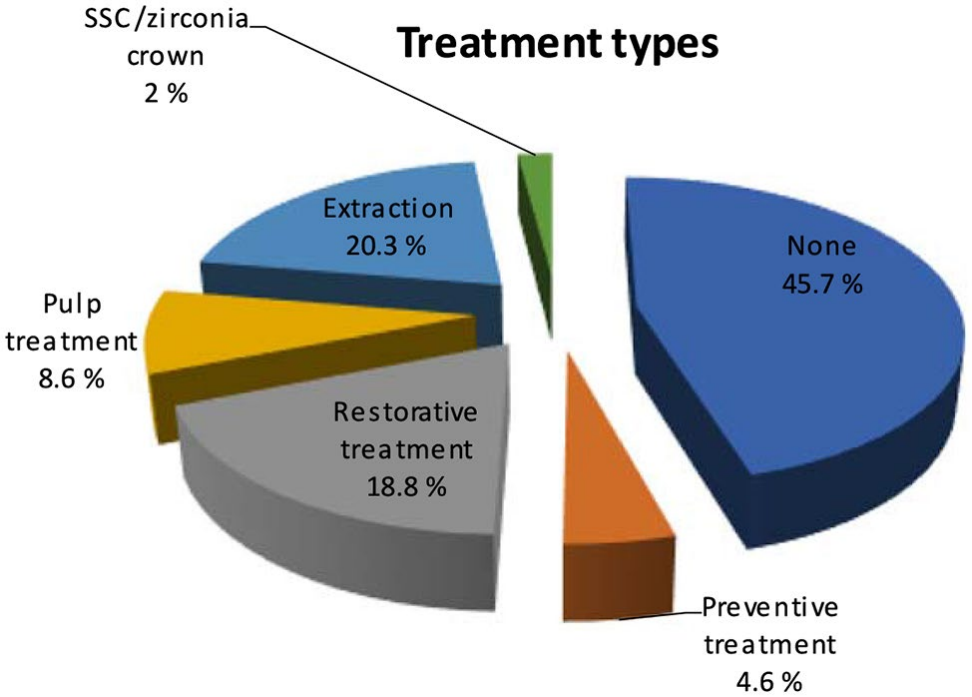
Data regarding the types of treatment performed on infraoccluded primary molars

While nearly half of the infraoccluded molars did not necessitate treatment, the frequencies of treatment types applied included extraction, restorative treatment, pulp treatment, preventive treatment, and application of SSC or zirconia crowns, respectively (Fig. 8).

The relationship between the classification of infraoccluded molars and the types of treatment applied is detailed in Table 4.

**Table 4:** Relationship between classification and treatment requirements in infraoccluded molars

	Group I	Group II	Group III	Group IV	
Treatment types	*n* (%)	*n* (%)	*n* (%)	*n* (%)	*p*
No intervention	81 (%49.1)	9 (%39.1)	0 (%0)	0 (%0)	0.001*
Preventive treatment	6 (%3.6)	2 (%8.7)	0 (%0)	0 (%0)	
Restorative treatment	35 (%21.2)	3 (%13)	0 (%0)	0 (%0)	
Pulp treatment	16 (%9.7)	0 (%0)	1 (%14.3)	0 (%0)	
Extraction	24 (%14.5)	8 (%34.8)	6 (%85.7)	2 (%100)	
SSC/zirconia crown	3 (%1.8)	1 (%4.3)	0 (%0)	0 (%0)	

Chi-Square test *p < 0.05

A statistically significant difference was observed concerning the classes of infraoccluded molars and the types of treatment applied (*p* < 0.05). Specifically, the rate of restorative treatment in Group I was significantly higher compared to other classes (*p* < 0.05), while extraction rates in Group III and Group IV were significantly higher than those in Group I and Group II (*p* < 0.05).

The frequency of accompanying findings associated with infraocclusion was as follows: tipping of adjacent teeth in 9.1%, significant deviation in midline shifts towards the affected side in 8.6%, increased caries in 7.6%, root resorption in 4.6%, and supraeruption of opposing tooth in 3.6%. Figures regarding the association between the number of infraoccluded teeth and accompanying findings are presented in Table 5.

**Table 5:** Evaluation of the relationship between the infraoccluded tooth number and accompanying findings

	54	55	64	65	74	75	84	85	
	*n* (%)	*n* (%)	*n* (%)	*n* (%)	*n* (%)	*n* (%)	*n* (%)	*n* (%)	*p*
Tipping of adjacent teeth (*n* = 18, %=9.1)	1 (%11.1)	3 (%25)	0 (%0)	2 (%18.2)	1 (%2.2)	8 (%38.1)	2 (%2.9)	1 (%4)	0.001*
Supraeruption of opposing tooth (*n* = 7, %=3.6)	1 (%11.1)	2 (%16.7)	0 (%0)	0 (%0)	1 (%2.2)	0 (%0)	1 (%1.4)	2 (%8)	0.101
Increase caries (*n* = 15, %=7.6)	0 (%0)	1 (%8.3)	0 (%0)	0 (%0)	4 (%8.9)	4 (%19)	4 (%5.8)	2 (%8)	0.629
Significant deviation in midline shift towards the affected side (*n* = 17, %=8.6)	2 (%22.2)	2 (%16.7)	1 (%20)	2 (%18.2)	3 (%6.7)	1 (%4.8)	3 (%4.3)	3 (%12)	0.146
Root resorption (*n* = 9, %=4.6)	1 (%11.1)	1 (%8.3)	0 (%0)	1 (%9.1)	2 (%4.4)	0 (%0)	4 (%5.8)	0 (%0)	0.523

Fisher Freeman Halton Exact test *p < 0.05

A statistically significant difference was observed in terms of the ‘tipping of adjacent teeth’ (p < 0.05). The incidence of ‘tipping of adjacent teeth’ in teeth numbered 55 (25%) and 75 (38.1%) was significantly higher compared to other teeth (*p* < 0.05).

Succeeding premolar germs were observed in 98% of infraoccluded molars. Table 6 displays the presence of succeeding premolar germs according to the class type in infraoccluded molars.

**Table 6:** Succeeding premolar germ presence according to the type of classification

		Presence of Successor tooth		
		Not present (*n* = 4, %=2)	Present (*n* = 193, %=98)	
		*n* (%)	*n* (%)	
Classification	Group I	2 (%1.2)	165 (%98.8)	0.029*
	Group II	1 (%4.8)	20 (%95.2)	
	Group III	0 (%0)	7 (%100)	
	Group IV	1 (%50)	1 (%50)	

Fisher Freeman Halton Exact test *p < 0.05

A statistically significant difference was observed in germ incidence rates between classes (*p* < 0.05). The germ ratio in Group IV teeth was significantly lower than in Group I, Group II, and Group III teeth. The location distribution of succeeding premolar teeth was as follows: 93.3% were positioned occlusally, 4.7% mesially, and 2.1% distally. A statistically significant relationship was observed between the location of the successor tooth germ beneath infraoccluded molars and the decision to extract (*p* < 0.05). The extraction rate of infraoccluded molars in which the successor tooth was occlusally located was significantly lower than those in which it was mesially or distally located (*p* < 0.05, Table 7).

**Table 7:** Association between location of successor tooth and extraction

Location of Successor tooth	Extraction		*p*
	Absent	Present	
	*n* (%)	*n* (%)	
**Oclusally** ***(n = 180, %=93.3)***	148 (%82.2)	32 (%17.8)	0.001*
**Mesially** ***(n = 9, %=4.7)***	4 (%44.4)	5 (%55.6)	
**Distally** ***(n = 4, %=2.1)***	2 (%50)	2 (%50)	

Fisher Freeman Halton Exact test *p < 0.05

## Discussion

A comprehensive retrospective analysis on a large group of subjects with infraocclusion of primary molars was carried out in this study. Associations between infraocclusion and factors such as age, gender, tooth type (primary first molar/primary second molar), arch type (maxillary/mandibular), and arch side (right/left) were scrutinized. The investigation encompassed an assessment of the prevalence, classification, diverse characteristics, and treatment modalities associated with infraoccluded primary molars. Additionally, the presence and the positioning of the succeeding premolar of the affected teeth were analyzed.

This study revealed a prevalence rate of 4.3% for infraocclusion in primary molars. A comparison with previous reports indicates variability in infraocclusion prevalence. Notably, published studies have reported prevalences of 8.9% among 1059 Swedish children aged 3–12 years [11], 24.8% among 1350 Israeli children aged 2.5–13.5 years [24], 1.3% among 2342 American schoolchildren of unspecified age [13], 10.48% among 849 children aged 3–12 years [25], 6.6% among 512 Italian children aged 5–15 years [26], 21.8% among 472 children aged 3–13 years [6], less than 1% in the maxilla and 22% in the mandible among 1454 singletons Finnish children, alongside 32% among 270 Australian twins aged 8–11 years [4], 3.25% among children from the West Mediterranean region of Turkey aged 7–11 years [5], and 7.38% among 542 Arabian children aged 4–12 years [3]. The diverse findings regarding infraocclusion prevalence across previous studies may be attributed to different styles of study data acquisition such as sample source and size, age range, diagnostic criteria.

The present study encompassed subjects aged 3 to 15 years and revealed that infraocclusion typically manifested at a mean age of 8.16 ± 1.71 years, consistent with recent systematic review [27] which verified the peak prevalence of infraocclusion at 8 to 9 years of age. The investigation established that infraocclusion most frequently occurred within the 6–9 years age range, followed by the 9–12 years age range, although instances have been observed in children as young as 4 years old. A previous study corroborated these findings, indicating that infraocclusion is predominantly documented between the ages of 6 and 11 years [19], aligning with the present study’s observations. Furthermore, upon examining the distribution of infraocclusion classes, it was noted that all severity levels (mild to extreme) were present among subjects aged 6–9 years (Table 2).

In the extant scientific literature, a multitude of classification systems [9, 19, 28] addressing infraocclusion are documented. Among these, Brearley et al. [9] and Kjaer et al. [19] stand as the two most prominently utilized schemes. Kjaer et al.‘s classification, illustrated in Fig. 1, delineates primary molar eruption by quantifying the distance from the occlusal plane of the primary molars to neighboring teeth in millimeters. In this investigation, the authors have opted for the latest classification system, Kjaer et al.‘s [19], to facilitate the study’s objectives.

In this investigation, no statistically significant difference was observed in the gender distribution among subjects with infraocclusion, which contrasts with findings from previous studies [3–5, 7]. Conversely, a recent study reported a higher incidence of infraocclusion among male children compared to female children [3]. The variation in results concerning gender-based distribution of infraocclusion prevalence remains a matter of ongoing debate and controversy.

Prior researches have indicated a higher prevalence of infraocclusion within the mandibular arch compared to the maxillary arch, with reported prevalence rates up to 10 times greater in the mandibular arch [5, 7, 26]. Additionally, previous studies have highlighted that mandibular molars are notably more affected by infraocclusion, with incidence rates reported to be up to 27 times higher compared to maxillary molars [4]. Consistent with these findings, the current study observed a higher frequency of mandibular infraocclusion compared to maxillary infraocclusion, with the most commonly affected teeth being the mandibular primary molars, specifically the lower right first primary molar, lower left first primary molar, and lower right second primary molar. While older reports [9, 29] have emphasized primary second molars as the primary affected teeth by infraocclusion, recent studies [4–6] have indicated that mandibular primary molars are the most affected, which aligns with the findings of the present study.

According to Mishra SK et al. when submerged primary teeth was observed it may cause malocclusion, late eruption of permanent teeth as a result of delayed root resorption [30]. Various factors contribute to the management of infraoccluded teeth, which are essential considerations when assessing patients presenting with this condition in general practice. Evaluation is necessary to ascertain if management can be effectively carried out in the general practice setting or if referral to a specialist is warranted. Factors influencing management encompass the presence or absence of a permanent successor, age of onset and severity of infraocclusion, rate of progression, adjacent teeth tipping, presence of other dental anomalies, and concurrent dental needs of the patient [23]. Clinical monitoring of mildly to moderately infraoccluded primary molars is recommended at three to six-month intervals, often necessitating no immediate intervention. In mild to moderate cases, it may be beneficial to restore the mesio-distal dimension and reconstruction of the occlusal plane height. This restoration can be achieved through the application of composite crowns or onlays, or potentially with the placement of a SSC without occlusal reduction [31]. Severe infraocclusion typically requires extraction due to adjacent teeth angulation resulting in space loss, successor teeth displacement and growth impediment, occlusal plane vertical disturbance, and escalating bone defects. Deciduous teeth lacking permanent successors demand particular attention. Post-extraction, space can be preserved or recreated using orthodontic appliances. Subsequently, the missing tooth can be replaced via autotransplantation or implantation, or the space can be closed during orthodontic treatment [32]. In the present study, when we looked at the classes while mild infraocclusion (Group I) cases resolved with restorative treatments, severe and extreme severe infraoccluded molars (Group III and Group IV) were needed extractions mostly. So, one may simply explain as the severity of the infraocclusion increases successful management may involve more invasive procedures.

Various manifestations are associated with infraocclusion, including delayed eruption of successor teeth with ectopic displacement, increased caries, periodontal diseases, root surface resorption, incomplete alveolar process development, lack of normal mesial drift, tipping of adjacent teeth, and overeruption of opposing teeth [33, 34]. Additionally, reductions in arch length and loss of space may be observed, particularly in cases of severe infraocclusion [35]. Notably, a significant deviation in midline shift towards the affected side may occur when the affected tooth remains in place, representing a notable clinical disturbance reported in the literature [36]. The present study evaluated the frequency of clinical disturbances accompanying infraocclusion, considering that five of the listed accompanying findings can be identified from orthopantomography. Among the three most common findings observed in the study population, tipping of adjacent teeth (9.1%) was the most frequent clinical disturbance, followed by significant deviation in midline shift towards the affected side (8.6%), and increased caries (7.6%). Previous research by Ersin et al. identified tipping of adjacent teeth as the most common manifestation of infraocclusion [15]. Similarly, Shalish et al. reported severe tilting of adjacent teeth crowns towards the affected area, with impacted premolars as a clinical manifestation [29]. Moreover, Peretz et al. noted that infraoccluded primary mandibular second molars were the most frequently tilted teeth [33], which is consistent with our results indicating a significantly higher incidence of the “tipping of adjacent teeth” finding in tooth numbers 55 and 75. Another commonly reported manifestation of infraoccluded teeth is an increased risk of caries in adjacent teeth and the infraoccluded tooth due to plaque accumulation and insufficient oral hygiene, attributed to difficult access to the area [37].

Some researchers suggested that if there was a successor tooth underneath and ankylosis was not detected, the infraoccluded tooth should not be extracted until the eruption time, however if ankylosis was detected, it should be extracted [15]. Sometimes alveolotomy may be useful for unerupted successor teeth. In cases with severe infraocclusion, the primary tooth with infraocclusion should be extracted and a space maintainer should be applied until the successor teeth erupt [14]. Another infrequently treatment alternative was luxation, by this way tooth can keep on its eruption. In that technique, the bone between the ankylosed tooth and alveolus was broken [38]. In cases where there was a loss of space due to the tipping of adjacent teeth, the lost space can be gained by applying an orthodontic appliance [23]. However; if the successor tooth was available and its thought to erupt lately, to avoid the tipping of the adjacent teeth, restorations to the occlusal surface or SSC were recommended [21, 37]. Briefly, a surgical management involving the extraction of the infraoccluded deciduous tooth in cases where the permanent successor was present. Only in cases in which permanent successor was diagnosed as absent was the choice made to preserve the infraoccluded deciduous tooth in the long term [27]. In the current investigation, agenesis of succeeding premolars was identified in 2% of infraoccluded molars. Notably, primary molars lacking successors exhibited more severe degrees of infraocclusion. Previous research conducted by Cardoso Silva et al. in a Spanish sample reported a prevalence of mandibular premolar agenesis at 3.8% among individuals presenting at least one infraoccluded molar [6]. Similarly, Zahit et al. documented a 21.78% incidence of agenesis of any permanent teeth in the context of infraocclusion [5]. A recent study has demonstrated an association between hypodontia and infraocclusion, with a frequency of 12.5% observed in an Arabian population [3]. While our findings align with those reported by Cardosa Silva et al. [6] the figures were lower than the prevalence rates reported in more recent investigations.

Kurol emphasized that progressive infraocclusion contributes to adjacent teeth tipping, bone defects, and impeded or delayed eruption of permanent successors, advocating for early removal, particularly when the permanent successor is in an incorrect position. Prior to extractions, consideration should be given to reopening or maintaining space [39]. Kurol further suggested that if the permanent successor is appropriately positioned, early extraction of the ankylosed deciduous molar may be unnecessary [40]. However, if there is an altered path of eruption for the successor tooth, extraction of the infraoccluded primary molar with a permanent successor should be considered. This finding is consistent with our observation that the extraction rate of infraoccluded molars with mesially and distally located successor teeth was higher than those with occlusally located successors.

The limitations of this study include the absence of quantitative measurements and long-term evaluation or follow-up of infraoccluded molars due to its retrospective design. Another noteworthy limitation pertains to the exclusion of orthodontic space opening procedures, as advocated by Shalissh et al. [29], subsequent to the extraction of severely infraoccluded deciduous teeth and the spontaneous eruption of impacted premolars. This exclusion results from the unavailability of pertinent data, thus limiting the comprehensive consideration of treatment types. However, a notable strength of this study is its investigation into the severity of infraocclusion across a broader range of age groups using a more recent classification system, which contributes to the current body of knowledge on this topic.

We highlight the crucial involvement of an interdisciplinary team in assessing and addressing infraocclusion, necessitating a spectrum of interventions customized to the severity and specific dental treatment needs of each patient. This comprehensive analysis seeks to advance understanding of infraocclusion, aiming to enhance patient outcomes by advocating for timely interventions and personalized therapeutic strategies.

## Conclusion

In conclusion, the prevalence of infraocclusion within the studied population was found to be 4.3%. This condition commonly emerges between the ages of 6 and 9 years, primarily affecting mandibular primary molars. Alongside infraocclusion, notable findings include tipping of adjacent teeth, substantial deviations in midline shifts towards the affected side, and increased susceptibility to caries. Furthermore, a subsequent premolar agenesis was identified in 2% of infraoccluded molars, with elevated extraction rates noted particularly in cases where the successor tooth was positioned mesially or distally. This study presents novel insights for dental practitioners, shedding light on the severity and distribution of necessary treatment interventions associated with infraocclusion. Notably, our findings suggest that as infraocclusion severity escalates, successful management may entail more invasive procedures, affirming our study hypothesis.

## Data Availability

The datasets used and/or analysed during the current study available from the corresponding author on reasonable request.

## Abbreviations

DAP: Dental anomaly patterns
SSC: Stainless-steel crowns
